# The Somatic Mutational Landscape of Mismatch Repair Deficient Prostate Cancer

**DOI:** 10.3390/jcm12020623

**Published:** 2023-01-12

**Authors:** Bangwei Fang, Yu Wei, Jian Pan, Tingwei Zhang, Dingwei Ye, Yao Zhu

**Affiliations:** 1 Department of Urology, Fudan University Shanghai Cancer Center, Shanghai 200032, China; 2 Department of Oncology, Shanghai Medical College, Fudan University, Shanghai 200032, China

**Keywords:** prostate cancer, mismatch repair-deficient, mutational landscape

## Abstract

Prostate cancers with mismatch repair deficiency (MMR-d) have aggressive clinical and histological features, and they are potentially responsive to immunotherapy. However, its rarity prevents the analysis of the underlying biology. Here, we collected the genomic data of 2664 primary prostate tumors and 1409 metastatic prostate tumors from the GENIE and TCGA databases. A total of 69 (2.59%) primary and 60 (4.26%) metastatic MMR-d tumors were identified among these tumors. Single nucleotide variant (SNV) frequencies of 34 candidate genes (including *KMT2D* (46.4%), *ZFHX3* (33.3%), *JAK1* (31.9%), and *RNF43* (27.5%)) and 16 candidate genes (including *KMT2D* (33.3%) and *JAK1* (28.3%)) were higher in MMR-d primary tumors and MMR-d metastatic tumors, respectively. The tumor mutation burden (TMB) was higher in primary MMR-d tumors. Homozygous deletions of *EPCAM* and *EPAS1* were enriched in MMR-d primary tumors, while *EPCAM* deletions were enriched in metastatic MMR-d tumors. For genomic rearrangement events, *TMPRSS2-ETS* fusions were less frequent in primary MMR-d tumors. Our study indicates MMR-d prostate cancers have unique genomic features. These may play an important role in providing therapeutic targets for the treatment of this subset of prostate cancer patients.

## 1. Introduction

DNA mismatch repair (MMR) is a mechanism for repairing incorrect base matches as well as insertion/deletion errors that occur during replication. MMR-deficient (MMR-d) status usually results from pathogenic alterations in the MMR core genes (*MLH1*, *MSH2*, *MSH6*, and *PMS2*), which are also associated with high microsatellite instability and high tumor mutational burden [1,2,3].

A prospective study indicated that men with *MSH2* or *MSH6* germline pathogenic mutations are at a significantly higher risk of prostate cancer (PCa) than non-carriers [4]. Several studies, however, have shown that MMR-d PCa are associated with aggressive histologic characteristics such as cribriform and ductal adenocarcinomas [5,6,7,8]. Furthermore, MMR-d PCa patients also have a high prevalence of Gleason ≥ 8, often present with de novo metastases, and respond poorly to androgen deprivation therapy (ADT) and next-generation antiandrogens [9,10,11]. Despite the fact that the presence of MMR-d status guides the use of immunotherapy, nearly half of these patients do not benefit from immunotherapy [12,13].

MMR-d is relatively rare in PCa, estimated at 3–5% of cases [11,12,14]. Due to their low prevalence, the molecular features of MMR-d PCa tumors remain incompletely described. We propose that MMR-d tumors represent a distinct phenotype in PCa. Recognition of genomic alterations in MMR-d PCa tumors may enable understanding of the mechanisms of MMR-d associated PCa carcinogenesis and progression, which may have the potential to provide downstream implications for treatment. In this study, we integrated the genomic data of primary and metastatic PCa from the TCGA and GENIE databases to depict the somatic mutational profiles in MMR-d prostate tumors and compare them with MMR-p tumors.

## 2. Materials and Methods

### 2.1. Samples and Data Processing

**Figure 1:**
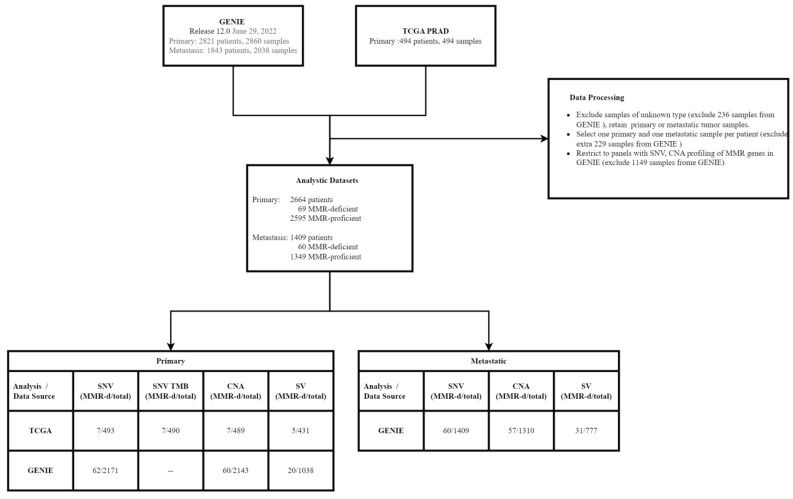
The workflow chart of the study. SNV = single nucleotide variant; TMB = tumor mutational burden; CNV = copy number variation; SV = structural variant; and MMR-d = mismatch repair-deficient.

The mutation files for SNV, CNA, and SV and clinical data from The Cancer Genome Atlas Prostate Adenocarcinoma (TCGA-PRAD) were retrieved from cBioportal (https://www.cbioportal.org/study/summary?id=prad_tcga_pan_can_atlas_2018, accessed on 12 September 2022). The SNV, CNA, and SV and clinical data from the American Association for Cancer Research (AACR) Project Genomics Evidence Neoplasia Information Exchange (GENIE) for prostate adenocarcinomas were downloaded from Sage Bionetworks Synapse platform release 12.0 (https://www.synapse.org, accessed on 12 September 2022). The GENIE data was filtered to retain only primary and metastatic samples with sequencing panels including SNV and CNA profiling of MMR genes (*MLH1*, *MSH2*, *MSH6*, and *PMS2*). The 14 retained panels were: COLU-CCCP-V1, DFCI-ONCOPANEL-1, DFCI-ONCOPANEL-2, DFCI-ONCOPANEL-3, DFCI-ONCOPANEL-3.1, MSK-IMPACT341, MSK-IMPACT410, MSK-IMPACT468, MSK-IMPACT505, UCSF-IDTV5-TO, UCSF-NIMV4-TO, VICC-01-DX1, VICC01-T5A, and VICC-01-T7. If a patient had multiple primary or metastatic samples, one sample was selected using the following hierarchy: (1) the sample was identified as a MMR-d tumor, (2) the sample had the maximal amount of available genomic data, and (3) if the first two criteria were not met, the sample was selected randomly. The sample sizes of the final analyzed population for primary and metastatic tumors by dataset and genomic data type are shown in Figure 1.

### 2.2. Identification of MMR-Deficient (MMR-d) Tumors

MMR-d status was determined by assessing the presence of SNV, CNA, and SV involving MMR genes in tumors. Pathogenic SNV of MMR genes were identified by the following process: (1) variants reported as oncogenic or likely oncogenic in OncoKB (http://oncokb.org, accessed on 12 September 2022) [15] were retained, and neutral variants were removed; and (2) the impact of the remaining variants with uncertain significance was further evaluated through SIFT and PolyPhen-2 [16,17]. Only variants classified as deleterious in SIFT and as probable or possibly damaging in PolyPhen-2 were retained. Tumors with pathogenic SNV were classified as MMR-d. However, tumors with homozygous deletions at MMR genes or SV affecting MMR genes were also classified as MMR-d.

### 2.3. Genomic Profiling Analyses

Tumor mutational burden (TMB) was calculated using SNV data from the TCGA database with whole-exome sequencing (WES) profiling. The capture size for the WES data was set to 50 Mbp. Differences in TMB by tumor MMR status were assessed. The candidate genes for analysis were identified from the literature [14,18,19], which included 14 hereditary genes in PCa, 7 gene interactors with MMR genes, and 27 genes recurrently mutated in PCa. In addition to this, we supplemented the top 20 genes with the highest SNV or CNV frequencies in primary or metastatic PCa samples from TCGA and GENIE. Since not all candidate genes were analyzed in the GENIE panels, the sample size for frequency calculations varied across genes. To ensure sufficient power, only genes profiled in at least 60% of tumors were studied further (Supplementary Table S1). The details of SNV, CNV, and SV analyses were presented in Supplementary Method.

## 3. Statistical Analysis

The frequencies of SNVs, CNAs, and SVs by MMR status were compared using the two-sided Fisher’s exact test. The Bonferroni’s correction was used to control the false discovery rate (FDR ≤ 1%).

## 4. Results

### 4.1. MMR-d Prostate Tumors

**Figure 2:**
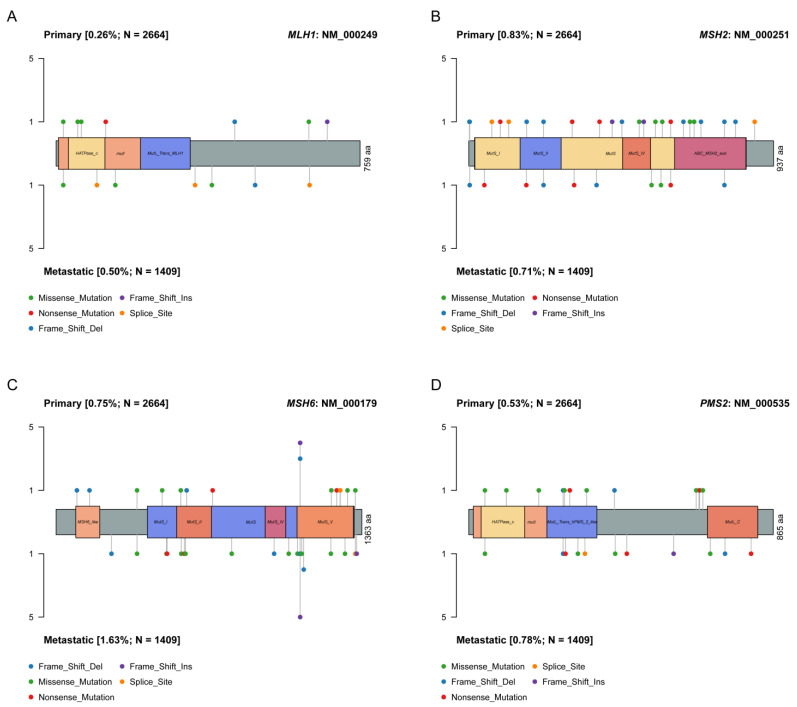
Locations of pathogenic mutations in MMR genes. Lollipop plots showing (**A**) pathogenic mutations of *MLH1*, (**B**) pathogenic mutations of *MSH2*, (**C**) pathogenic mutations of *MSH6* and (**D**) pathogenic mutations of *PMS2*. The mutations in primary tumors are displayed above the lollipop, and the mutations in metastatic tumors are displayed below the lollipop. aa = amino acid; Del = deletion; Ins = insertion.

The genomic data of 2664 primary and 1409 metastatic prostate adenocarcinomas was collected from the American Association for Cancer Research Project Genomics Evidence Neoplasia Information Exchange (GENIE) and The Cancer Genome Atlas (TCGA) databases (Figure 1). A total of sixty-nine (2.59%) primary tumors harbored pathogenic SNVs, CNAs, or SVs leading to MMR-d, while 2595 tumors (97.41%) did not have pathogenic alterations at MMR genes and were classified as MMR-p. Seven tumors had pathogenic SNVs at *MLH1*, twenty-two tumors had pathogenic SNVs at *MSH2*, twenty tumors had pathogenic SNVs at *MSH6,* and fourteen tumors had pathogenic SNVs at *PMS2* (Figure 2). A total of one tumor had homozygous deletion at *MLH1*, two tumors had homozygous deletion at *PMS2*, two tumors had homozygous deletion at *MSH2*, one tumor had homozygous deletion at *MSH6,* and seven tumors had homozygous deletion at both *MSH2* and *MSH6*. A total of two tumors had SVs affecting *MLH1*, two tumors had SVs affecting *MSH2,* and three tumors had SVs affecting *MSH6* (Supplementary Table S2).

A total of 60 (4.26%) metastatic tumors were identified as MMR-d, while 1349 tumors (97.41%) were MMR-p. A total of seven tumors had pathogenic SNVs at *MLH1*, ten tumors had pathogenic SNVs at *MSH*2, twenty-three tumors had pathogenic SNVs at *MSH6,* and eleven tumors had pathogenic SNVs at *PMS2* (Figure 2). A total of two tumors had homozygous deletion at *MLH1*, three tumors had homozygous deletion at *MSH2*, two tumors had homozygous deletion at *MSH6,* and eight tumors had homozygous deletion at both *MSH2* and *MSH6*. A total of one tumor had SVs affecting *MLH1*, five tumors had SVs affecting *MSH2,* and two tumors had SVs affecting *MSH6* (Supplementary Table S3).

### 4.2. Single Nucleotide Variant (SNV) Analyses

**Figure 3:**
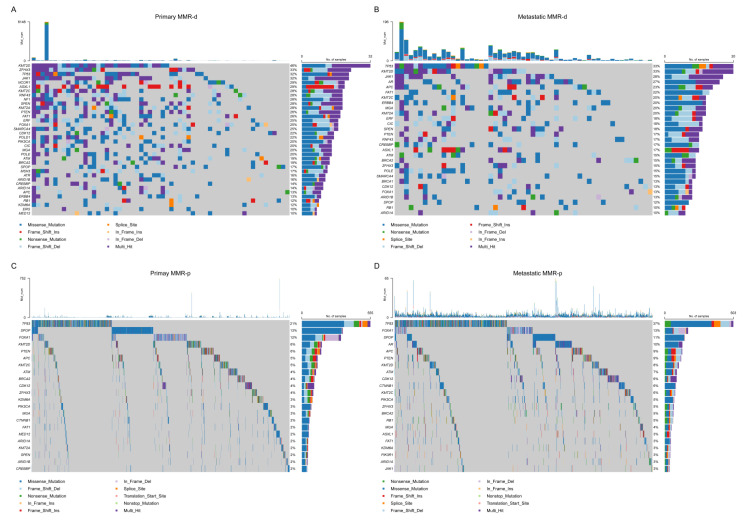
Oncoplot of single nucleotide variants in primary and metastatic MMR-d and MMR-p tumors. Plots correspond to (**A**) primary mismatch repair-deficient tumors, (**B**) metastatic mismatch repair-deficient tumors, (**C**) primary mismatch repair-proficient tumors, and (**D**) metastatic mismatch repair-proficient tumors. Only the most frequently altered candidate genes are shown in the figure. MMR-d = mismatch repair-deficient; MMR-p = mismatch repair-proficient; Del = deletion; Ins = insertion.

**Figure 4:**
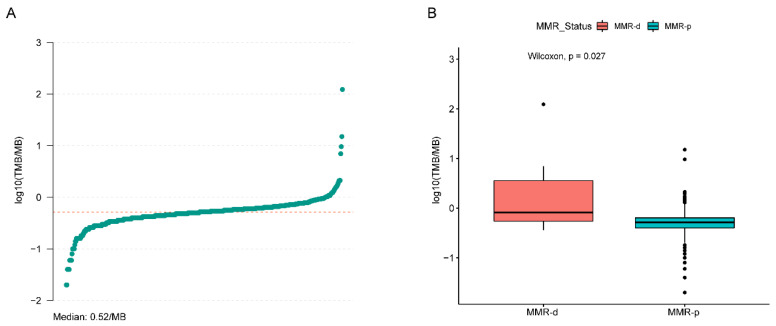
Tumor mutational burden in primary MMR-d and MMR-p tumor samples. (**A**) The distribution of TMB in all tumors. (**B**) The distribution of TMB by MMR status. The *Y*-axis represents the log10-transformed tumor mutation burden per megabase. TMB = tumor mutational burden; MMR-d = mismatch repair-deficient; and MMR-p = mismatch repair-proficient.

The most frequent SNVs of candidate genes in MMR-d and MMR-p primary tumors derived by combining TCGA and GENIE data are shown in Figure 3a,c. The SNV frequency of 34 candidate genes in MMR-d primary tumors was significantly higher than in MMR-p primary tumors. A total of 19 of these genes had a SNV frequency ≥20% in MMR-d tumors. The most significant differences by MMR status were found in *JAK1* (31.9% vs. 1.5%, Bonferroni-adjusted *p* = 2.1 × 10^−21^), *ASXL1* (29.0% vs. 1.0%, Bonferroni-adjusted *p* = 5.6 × 10^−21^), *KMT2D* (46.4% vs. 6.2%, Bonferroni-adjusted *p* = 2.1 × 10^−19^), and *RNF43* (27.5% vs. 1.08%, Bonferroni-adjusted *p* = 1.1 × 10^−17^) (Supplementary Table S4). For metastatic tumors from GENIE, a total of sixteen candidate genes had significantly higher SNV frequencies in MMR-d tumors, of which six genes (*KMT2D* (33.3%), *JAK1* (28.3%), *FAT1* (21.7%), *ERBB4* (20.0%), *KMT2A* (20.0%), and *MGA* (20.0%)) had a SNV frequency ≥ 20% in MMR-d tumors. *TP53* had the highest SNV frequency in both MMR-d and MMR-p tumors, but its frequency did not differ between MMR-d (33.3%) and MMR-p (37.3%) metastatic tumors (*p* = 0.56) (Figure 3b,d, Supplementary Table S5). Among 490 patients with pmary tumors from the TCGA database, the median SNV TMB was 0.52 per MB (range: 0.02–122.96) (Figure 4a). SNV TMB was significantly higher in MMR-d tumors than in MMR-p tumors (median: 0.82 vs. 0.52, *p* = 0.024) (Figure 4b).

### 4.3. Copy Number Alteration (CNA) Analyses

**Figure 5:**
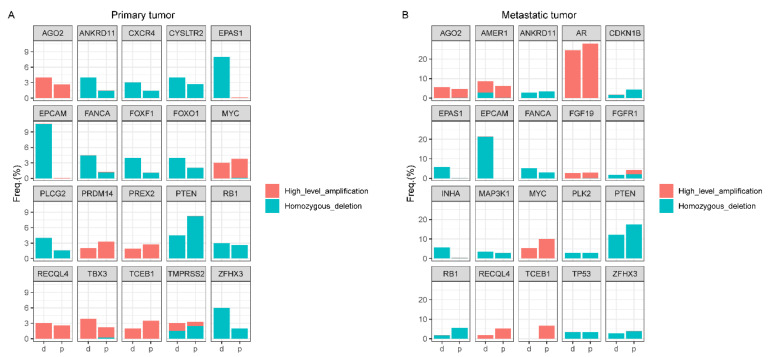
Copy number variant frequency in MMR-d and MMR-p tumors. Plots correspond to (**A**) primary and (**B**) metastatic tumors. Only the most frequently altered candidate genes are shown in the figure. MMR-d = mismatch repair-deficient; MMR-p = mismatch repair-proficient; d = mismatch repair-deficient; p = mismatch repair-proficient.

For primary tumors from the TCGA and GENIE databases, homozygous deletions at *EPCAM* (10.6% vs. 0%, Bonferroni-adjusted *p* = 2.6 × 10^−10^) and *EPAS1* (8.0% vs. 0.05%, Bonferroni-adjusted *p* = 7.0 × 10^−5^) were significantly more frequent in MMR-d tumors (Figure 5a, Supplementary Table S6). Among metastatic tumors from the GENIE database, the frequency of homozygous deletions at *EPCAM* (21.4% vs. 0%, Bonferroni-adjusted *p* = 4.2 × 10^−16^) was significantly higher in MMR-d tumors (Figure 5b, Supplementary Table S7). There were no other gene differences in homozygous deletion frequencies by MMR status after multiple testing correction. The homozygous deletion frequency at *PTEN* was non-significantly lower in MMR-d tumors than in MMR-p tumors (primary: 4.5% vs. 8.2%, metastatic: 12.3% vs. 17.6%). The frequencies of homozygous deletions at *ETV1* (3.0% vs. 0.1%, *p* = 0.0037), *KMT2C* (3.9% vs. 0.19%, *p* = 0.0076), and *FANCA* (4.8% vs. 1.17%, *p* = 0.0499) were nominally higher in MMR-d primary tumors, as were the frequencies of homozygous deletions at *EPAS*1 (5.7% vs. 0.1%, *p* = 0.0030) and *INHA* (5.7% vs. 0.3%, *p* = 0.0094) in MMR-d metastatic tumors. Nevertheless, there was no significant difference in these frequencies after FDR correction.

No significant differences in the frequency of genes with high-level amplification were found between MMR-d and MMR-p tumors, both in primary tumors and metastatic tumors. The high-level amplification frequencies at *MYC* (primary: 3.0% vs. 3.7%, metastatic: 5.3% vs. 10.0%) and *AR* (primary: 0% vs. 1.3%, metastatic: 24.6% vs. 27.9%) were non-significantly lower in MMR-d tumors than MMR-p tumors (Supplementary Tables S8 and S9).

### 4.4. Structural Variant (SV) Analyses

For primary tumors, the frequencies of *TMPRSS2*- and *ETS*-related SVs were evaluated using the TCGA and GENIE databases. *TMPRSS2*-*EST* fusions were less common in MMR-d primary tumors than MMR-p primary tumors (32.0% vs. 54.2%, *p* = 0.041). The frequencies of *TMPRSS2*-*ERG*, *TMPRSS2*-intragenic, and other SVs affecting *ETS* genes were insignificantly less in MMR-d tumors than in MMR-p tumors. As for metastatic tumorsrom the GENIE database, the prevalence of *TMPRSS2*- and *ETS*-related SVs did not differ by MMR status (Table 1).

## 5. Discussion

MMR-d status was not common in PCa patients. However, they have attracted the attention of clinicians since the FDA granted approval for the PD-1 inhibitor pembrolizumab for the treatment of MMR-d tumors regardless of the cancers’ tissue of origin in May 2017 [20]. Immunotherapy has shown promising efficacy in MMR-d PCa patients, but there is growing evidence that PD-1 inhibitors are not effective in all MMR-d patients, and some MMR-p patients are also sensitive to immunotherapy [12,21]. In addition, it was suggested that MMR-d status could also lead to *BRCA1/2* mutations, but these concurrent *BRCA1/2* mutations do not lead to PARP-inhibitor sensitivity [22]. To improve the understanding of MMR-d in PCa, our work provides integrated analyses of the genomic landscape in primary and metastatic prostate tumors.

In our study, we found that the TMB in MMR-d tumors was significantly higher than in MMR-p tumors in the context of a low overall TMB in PCa, which is consistent with previous findings [10,11]. However, despite a significant increase in the mutation frequencies of most candidate genes in MMR-d tumors, we found no significant increase in the frequency of the most common genomic aberrations in PCa driver genes, such as *TP53*, *FOXA1*, and *SPOP*. In addition, other molecular events such as *PTEN* deletion, *RB1* deletion, and *MYC* amplification were less common in MMR-d tumors, although they did not reach a significant difference. While these genomic aberrations usually indicate highly aggressive tumors as well as a poor prognosis, MMR-d tumors had a lower incidence of these molecular events yet still exhibited a highly malignant biology and responded poorly to ADT and next-generation antiandrogens [10,11]. These results suggest that MMR-d PCa tumors possess a unique molecular profile, and the development of this group of tumors may not be dependent on the common tumor pathways activated in PCa.

Notably, we observed that *KMT2D*, also known as *MLL2*, mutated in close to half of MMR-d primary tumors and in 1/3 of MMR-d metastatic tumors. *KMT2D* encodes a histone methyltransferase that methylates the Lys-4 position of histone H3, and it plays an important role in DNA damage repair. Mutations in *KMT2D* occur widely in numerous types of tumors and have been proven to be directly associated with genomic instability [23,24]. It is worth noting that a recent study showed that *KMT2D*-mutant tumors exhibit enhanced immune infiltration in the tumor microenvironment, and patients with *KMT2D* mutations are sensitive to immunotherapy [25]. Combined with clinical data indicating that nearly half of MMR-d prostate cancer patients are sensitive to immunotherapy [12,13], a proportion similar to the proportion of *KMT2D* mutations in MMR-d tumors. Therefore, we speculate that the high mutation rate in *KMT2D* may be partly accountable for the favorable efficacy of immunotherapy in MMR-d PCa patients, and the identification of *KMT2D* mutations may have important implications for further subdividing the population of PCa patients who can benefit from immunotherapy. In addition, mutations in *KMT2D* in PCa were also shown to be associated with aggressive biologic behaviors as well as a poor prognosis [26], which was consistent with the clinical features of MMR-d PCa patients, suggesting that *KMT2D* mutations may be engaged in the carcinogenesis and progression of MMR-d tumors and that it might be a potential therapeutic target for MMR-d PCa patients. In addition, we found that the SNV frequency at *JAK1* was also significantly increased among MMR-d tumors. *JAK1* encodes a membrane protein that is a member of a group of protein tyrosine kinases (PTKs). The encoded kinase phosphorylates STAT proteins and plays a crucial role in affecting the expression of genes that mediate inflammation, epithelial remodeling, and metastatic cancer progression. It is a pivotal constituent of the interleukin-6 (IL-6)/JAK1/STAT3 immune and inflammation response and is a therapeutic target for alleviating cytokine storms [27]. More importantly, there is evidence that loss-of-function mutations at *JAK1* promote a lack of response to interferon gamma, leading to resistance to immunotherapy in tumor cells [28]. Therefore, mutations in *JAK1* may contribute to primary resistance to immunotherapy in some PCa patients with MMR-d. However, it is noteworthy that Laura et al. reported that in MMR-d PCa patients, the mutations of *JAK1* did not associate with diminished efficacy of immunotherapy, Hence, the *JAK1* mutations may be passenger mutations occurring in MMR-d PCa patients, which, like genes containing microsatellite tracts (such as *RNF43*), have a higher propensity for secondary mutations in MMR-d tumors [29]. We also analyzed the four MMR genes separately and found that mutations in some genes, like *KMT2D* and *FAT1,* were mainly enriched in tumors carrying deleterious aberrations in *MSH2* and *MSH6,* which reflect that tumors carrying different MMR gene aberrations may have different genomic features. Given the low number of tumor specimens analyzed per individual MMR gene, we plan to validate these findings in a larger cohort of samples. Furthermore, considering that we do not currently treat patients carrying different MMR gene mutations differently in clinical decision-making, we believe it is reasonable to have them grouped together for the present analysis. Combining these results, we believe that a comprehensive assessment of the genomic characteristics of PCa patients is necessary before the administration of immunotherapy.

We also found higher frequencies of homozygous deletions for *EPCAM* and *EPAS1* in MMR-d primary tumors and for *EPCAM* in MMR-d metastatic tumors. *EPCAM* encodes a carcinoma-associated antigen, which is being used as a target for immunotherapy treatment [30]. It is located upstream of *MSH2,* and the germline deletions at *EPCAM* can cause *MSH2* epigenetic inactivation, leading to Lynch syndrome in the absence of *MSH2* pathogenic alterations [31,32]. Considering that *EPCAM* deletions have a relatively high frequency in MMR-d tumors (21.4% in MMR-d metastatic tumors), we presume that the prevalence of Lynch syndrome in MMR-d prostate cancer may be higher than previously thought [33].

Genomic alterations in *ETS*-related genes (mainly *ERG*) due to a fusion between the androgen receptor regulatory gene promoter of *TMPRSS2* and the *ETS* transcription factors are detected in approximately half of PCa tumors [34]. Several studies have indicated that *TMPRSS2*-*ETS* fusions perform a critical role in PCa tumorigenesis [35,36]. However, in our study, we found that MMR-d tumors had a lower incidence of *TMPRSS2*-*ETS* fusions relative to MMR-p tumors, which is consistent with previous findings in a small MMR-d PCa cohort [5]. It suggests that the mechanism of tumorigenesis in MMR-d PCa tumors is likely different from that of most PCa tumors.

To the best of our knowledge, our analysis has represented the largest primary and metastatic MMR-d prostate tumors to date and investigated the characteristics of multi-class somatic mutations. Although we identified possible associations between the characteristic somatic mutations in MMR-d PCa tumors and the efficacy of immunotherapy, there are still some limitations in our study. Firstly, although more than 90% of MMR-d tumors are caused by somatic mutations, germline mutations in MMR genes and *EPCAM* account for 5–10% of dMMR tumors, which are also known as Lynch syndrome [37]. Thus, we may have misclassified a small portion of MMR-d tumors as MMR-p tumors. This misclassification may mitigate the differences in the pattern of alterations between MMR-d and MMR-p tumors. Second, since the GENIE database did not provide detailed clinical data on patients, we were unable to compare the clinical characteristics among patients with MMR-d PCa tumors and those with MMR-p PCa tumors. Third, due to the relatively low proportion of MMR-d status in PCa, the limited number of MMR-d tumor specimens restricts us from doing further analysis by classifying different MMR gene mutations, and the genomic alterations do not fully reflect the DNA repair capacity. In addition, the inclusion of patients with potentially non-pathogenic MMR missense mutations might have diluted our findings. However, we only retained variants reported as oncogenic or likely oncogenic in OncoKB (http://oncokb.org, accessed on 12 September 2022) and removed variants unless they were classified as deleterious in SIFT and as probably damaging or possibly damaging in PolyPhen-2 (as detailed in the Methods section). With this strategy, >70% of missense mutations involving MMR genes were filtered, and the percentage of MMR-d tumors (2.59% in primary tumors and 4.26% in metastatic tumors) is consistent with previous studies (3–5%) [11,12,14]. If all missense mutations were removed, then only 1.6% of primary tumors and 2.7% of metastatic tumors would be classified as MMR-d tumors, a significantly lower proportion than reported in previous studies.

## 6. Conclusions

In conclusion, our study reveals the unique somatic mutational landscape of MMR-d PCa and provides partial evidence from this perspective to explain why this population is potentially sensitive to immunotherapy. These results also provide information for the development of therapeutic targets and clinical management for this group of patients.

## Figures and Tables

**Table 1:** Structural variant (SV) frequency by MMR status for primary and metastatic prostate tumors.

SV Type	Primary MMR-d	Primary MMR-p	*p*-Value	Metastatic MMR-d	Metastatic MMR-p	*p*-Value
*TMPRSS2*-*ETS*						
Present	8 (32.0%)	783 (54.2%)	0.041	11 (35.5%)	360 (48.3%)	0.200
Absent	17 (68.0%)	661 (45.8%)		20 (64.5%)	386 (51.7%)	
*TMPRSS2*-*ERG*						
Present	8 (32.0%)	755 (52.3%)	0.067	10 (32.3%)	353 (47.3%)	0.141
Absent	17 (68.0%)	689 (47.7%)		21 (67.7%)	393 (52.7%)	
*ETS*-others						
Present	0 (0%)	62 (4.3%)	0.622	1 (3.2%)	22 (2.9%)	0.613
Absent	25 (100%)	1382 (95.7%)		30 (96.8%)	724 (97.1%)	
*ERG*-others						
Present	0 (0%)	32 (2.2%)	1.000	1 (3.2%)	6 (0.8%)	0.249
Absent	25 (100%)	1412 (97.8%)		30 (96.8%)	740 (99.2%)	
*TMPRSS2*-intragenic						
Present	1 (5.0%)	75 (7.4%)	1.000	1 (3.2%)	54 (7.2%)	0.718
Absent	19 (95.0%)	943 (92.6%)		30 (96.8%)	692 (92.8%)	

*p*-values were from the two-sided Fisher’s exact test. MMR = mismatch repair; MMR-d = mismatch repair-deficient; MMR-p = mismatch repair-proficient.

## Data Availability

The data of this study are available from the AACR Project GENIE database (https://www.synapse.org/#!Synapse:syn7222066/wiki/410924, accessed on 12 September 2022) and The Cancer Genome Atlas (https://www.cbioportal.org/study/summary?id=prad_tcga_pan_can_atlas_2018, accessed on 12 September 2022).

## Supplementary Materials

The following supporting information can be downloaded at: https://www.mdpi.com/article/10.3390/jcm12020623/s1, Supplementary Method: Supplementary method for genomic profiling analyses [14,18,19,38]; Table S1: Candidate Gene Genlist for analysis; Table S2: List of MMR-d primary tumors; Table S3: List of MMR-d metastatic tumors; Table S4: SNV frequency by MMR status in primary tumors; Table S5: SNV frequency by MMR status in metastatic tumors; Table S6: CNV frequency by MMR status in primary tumors (Homozygous Deletion); Table S7: CNV frequency by MMR status in metastatic tumors (Homozygous Deletion); Table S8: CNV frequency by MMR status in primary tumors (High level of amplification); Table S9: CNV frequency by MMR status in metastatic tumors (High level of amplification).
