# Supplementary material for: The Somatic Mutational Landscape of Mismatch Repair Deficient Prostate Cancer
Source: J Clin Med. 2023 Jan 12;12(2):623. doi: 10.3390/jcm12020623 (PMC9864094; doi:10.3390/jcm12020623)
Supplement: Supplementary file 1 [file jcm-12-00623-s001.zip › Supplementary Method.pdf]

Supplementary method for genomic profiling analyses

Single nucleotide variant (SNV) analyses

SNV data were converted to maf format using maftools [1]. SNV frequency was determined as the percentage of individuals who were analyzed for a particular gene that carried a variant affecting the gene of interest. 48 Candidate genes including 14 hereditary genes in PCa, 7 genes interactors with MMR genes and 27 genes recurrently mutated in PCa were determined from the literature [2-4] for inclusion in the somatic mutation analyses. The top 20 genes with the highest frequency of SNVs in primary or metastatic tumors were added to the candidate genes (Supplementary Table S2). Since not all candidate genes were analyzed in the GENIE panels, the sample size for frequency calculations varied across genes. To assure sufficient power, only genes profiled in at least 60% of tumors were continued to be analyzed.

Copy number alteration analysis

CNA frequencies were defined as the percentage of individuals who were analyzed for a particular gene that carried a CNA affecting the gene of interest. Candidate genes for CNA analyses were identified in a similar manner as for the SNV analyses. 48 genes identified in the literature were supplemented with genes among the top 20 most commonly copy number-altered in primary or metastatic tumors and only genes profiled in at least 60% of tumors were continued to be analyzed., resulting in 61 candidate genes for primary tumors and 59 candidate genes for metastatic tumors (Supplementary Table S2).

***TMPRSS2* and *ETS* structural variant (SV) analyses**

Alteration frequencies were calculated for harboring any SV affecting *TMPRSS2*, *ERG*, or *ETS* family genes, accounting for gene coverage across the GENIE panels. Samples with SV profiling for at least one *ETS* family gene were included in estimating frequencies of *ETS* family SVs. *ETS* genes considered included: *ERG*, *ETV1*, *ETV4* and *ETV*. Other genes in *ETS* family were not included due to the extremely low frequencies of SV in PCa. SVs of interest were classified as *TMPRSS2-ETS*, *TMPRSS2-ERG* *ETS*-other, *ERG*-others and *TMPRSS2*-intragenic. Differences in alteration frequencies for these 5 SV categories by MMR status were tested.

References

1. Mayakonda A, Lin DC, Assenov Y, Plass C, Koeffler HP. Maftools: efficient and comprehensive analysis of somatic variants in cancer. Genome Res 2018;28: 1747-56.

2. Robinson D, Van Allen EM, Wu YM, Schultz N, Lonigro RJ, Mosquera JM, Montgomery B, Taplin ME, Pritchard CC, Attard G, Beltran H, Abida W, et al. Integrative clinical genomics of advanced prostate cancer. Cell 2015;161: 1215-28.

3. Cancer Genome Atlas Research N. The Molecular Taxonomy of Primary Prostate Cancer. Cell 2015;163: 1011-25.

4. Mao R, Krautscheid P, Graham RP, Ganguly A, Shankar S, Ferber M, Hegde M, Committee ALQA. Genetic testing for inherited colorectal cancer and polyposis, 2021 revision: a technical standard of the American College of Medical Genetics and Genomics (ACMG). Genet Med 2021;23: 1807-17.
