# Supplementary material for: The Somatic Mutational Landscape of Mismatch Repair Deficient Prostate Cancer
Source: J Clin Med. 2023 Jan 12;12(2):623. doi: 10.3390/jcm12020623 (PMC9864094; doi:10.3390/jcm12020623)
Supplement: Supplementary file 1 [file jcm-12-00623-s001.zip › Supplementary Tables.pdf]

Supplementary Table S2. List of MMR-d primary tumors

**Patient ID**	**Sample ID**	**Gene**	**Alteration Type**	**Alteration Description**
TCGA-EJ-7782	TCGA-EJ-7782-01	*MSH2*	SNV	N538Kfs*3
TCGA-EJ-A65B	TCGA-EJ-A65B-01	*MLH1*	SNV	R18C
TCGA-XK-AAIW	TCGA-XK-AAIW-01	*MSH6*	SNV	F582Lfs*4
TCGA-XK-AAIW	TCGA-XK-AAIW-01	*PMS2*	SNV	R107Q
GENIE-MSK-P-0004387	GENIE-MSK-P-0004387-T03-IM6	*MSH6*	SNV	Y556H
GENIE-MSK-P-0004946	GENIE-MSK-P-0004946-T01-IM5	*PMS2*	SNV	R287*
GENIE-DFCI-004303	GENIE-DFCI-004303-3443	*PMS2*	SNV	N335S
GENIE-DFCI-004161	GENIE-DFCI-004161-4096	*MSH6*	SNV	I1227V
GENIE-MSK-P-0015570	GENIE-MSK-P-0015570-T01-IM6	*MLH1*	SNV	C124*
GENIE-MSK-P-0015837	GENIE-MSK-P-0015837-T01-IM6	*MLH1*	SNV	D63G
GENIE-MSK-P-0008387	GENIE-MSK-P-0008387-T01-IM5	*MSH6*	SNV	F1088Lfs*5
GENIE-MSK-P-0009892	GENIE-MSK-P-0009892-T01-IM5	*MSH6*	SNV	G93Efs*56
GENIE-MSK-P-0009892	GENIE-MSK-P-0009892-T01-IM5	*PMS2*	SNV	R199H
GENIE-DFCI-002078	GENIE-DFCI-002078-10200	*PMS2*	SNV	D414Tfs*34
GENIE-DFCI-009654	GENIE-DFCI-009654-8308	*MSH2*	SNV	A2T
GENIE-DFCI-011099	GENIE-DFCI-011099-11350	*PMS2*	SNV	R646M
GENIE-DFCI-051036	GENIE-DFCI-051036-203414	*MSH2*	SNV	A714Lfs*6
GENIE-DFCI-039653	GENIE-DFCI-039653-233142	*MSH6*	SNV	X1267_splice
GENIE-DFCI-028582	GENIE-DFCI-028582-158650	*MLH1*	SNV	D631Y
GENIE-DFCI-090897	GENIE-DFCI-090897-350665	*MLH1*	SNV	S677Tfs*107
GENIE-MSK-P-0021894	GENIE-MSK-P-0021894-T01-IM6	*MSH2*	SNV	E786Dfs*11
GENIE-MSK-P-0022120	GENIE-MSK-P-0022120-T01-IM6	*MSH2*	SNV	C693R
GENIE-MSK-P-0022120	GENIE-MSK-P-0022120-T01-IM6	*MSH6*	SNV	F1088Lfs*5
GENIE-MSK-P-0023307	GENIE-MSK-P-0023307-T01-IM6	*MSH6*	SNV	F1088Lfs*5
GENIE-MSK-P-0023405	GENIE-MSK-P-0023405-T01-IM6	*MSH2*	SNV	Q402*
GENIE-MSK-P-0023405	GENIE-MSK-P-0023405-T01-IM6	*MSH2*	SNV	L595P
GENIE-MSK-P-0026871	GENIE-MSK-P-0026871-T01-IM6	*MSH6*	SNV	L473V
GENIE-MSK-P-0027380	GENIE-MSK-P-0027380-T01-IM6	*MSH2*	SNV	X71_splice
GENIE-MSK-P-0028654	GENIE-MSK-P-0028654-T01-IM6	*PMS2*	SNV	F272C
GENIE-MSK-P-0029127	GENIE-MSK-P-0029127-T01-IM6	*MSH6*	SNV	K149Nfs*25
GENIE-MSK-P-0029164	GENIE-MSK-P-0029164-T01-IM6	*MSH2*	SNV	K471Nfs*11
GENIE-MSK-P-0030465	GENIE-MSK-P-0030465-T01-IM6	*MSH2*	SNV	X879_splice
GENIE-DFCI-123573	GENIE-DFCI-123573-1149676	*MSH6*	SNV	F1088Lfs*5
GENIE-DFCI-106275	GENIE-DFCI-106275-804214	*PMS2*	SNV	S46I
GENIE-MSK-P-0032288	GENIE-MSK-P-0032288-T01-IM6	*PMS2*	SNV	Y268Tfs*39
GENIE-MSK-P-0032701	GENIE-MSK-P-0032701-T01-IM6	*MSH2*	SNV	V3Dfs*74
GENIE-MSK-P-0033077	GENIE-MSK-P-0033077-T01-IM6	*MSH6*	SNV	G1299D
GENIE-MSK-P-0034070	GENIE-MSK-P-0034070-T01-IM6	*MSH2*	SNV	R524C
GENIE-MSK-P-0034070	GENIE-MSK-P-0034070-T01-IM6	*MSH6*	SNV	F1088Sfs*2
GENIE-MSK-P-0035133	GENIE-MSK-P-0035133-T01-IM6	*MSH6*	SNV	F1088Sfs*2

GENIE-MSK-P-0035133	GENIE-MSK-P-0035133-T01-IM6	*PMS2*	SNV	E656*
GENIE-MSK-P-0035426	GENIE-MSK-P-0035426-T01-IM6	*MSH6*	SNV	S1251*
GENIE-MSK-P-0035426	GENIE-MSK-P-0035426-T01-IM6	*MSH6*	SNV	R1334W
GENIE-MSK-P-0035426	GENIE-MSK-P-0035426-T01-IM6	*PMS2*	SNV	R665I
GENIE-MSK-P-0036805	GENIE-MSK-P-0036805-T01-IM6	*MSH6*	SNV	F1088Sfs*2
GENIE-MSK-P-0040259	GENIE-MSK-P-0040259-T01-IM6	*MSH2*	SNV	R621*
GENIE-MSK-P-0041356	GENIE-MSK-P-0041356-T01-IM6	*MSH2*	SNV	X123_splice
GENIE-MSK-P-0043317	GENIE-MSK-P-0043317-T01-IM6	*PMS2*	SNV	R287Sfs*19
GENIE-MSK-P-0043520	GENIE-MSK-P-0043520-T01-IM6	*MSH2*	SNV	Q97*
GENIE-VICC-155853	GENIE-VICC-155853-unk-1	*MSH2*	SNV	R680P
GENIE-DFCI-261954	GENIE-DFCI-261954-3646368	*MSH2*	SNV	E318*
GENIE-DFCI-268567	GENIE-DFCI-268567-3733748	*MSH2*	SNV	Q574P
GENIE-MSK-P-0054354	GENIE-MSK-P-0054354-T01-IM6	*MSH2*	SNV	D660Ifs*25
GENIE-MSK-P-0052998	GENIE-MSK-P-0052998-T01-IM6	*PMS2*	SNV	P654T
GENIE-MSK-P-0055256	GENIE-MSK-P-0055256-T01-IM6	*MLH1*	SNV	G54V
GENIE-UCSF-12162	GENIE-UCSF-12162-2479T	*MSH6*	SNV	X1334_splice
GENIE-MSK-P-0059427	GENIE-MSK-P-0059427-T01-IM7	*MSH2*	SNV	F178Sfs*36
GENIE-MSK-P-0059427	GENIE-MSK-P-0059427-T01-IM7	*MSH6*	SNV	R361H
GENIE-MSK-P-0061209	GENIE-MSK-P-0061209-T01-IM7	*PMS2*	SNV	R107W
GENIE-MSK-P-0062920	GENIE-MSK-P-0062920-T01-IM7	*MSH6*	SNV	H1266Y
GENIE-MSK-P-0064623	GENIE-MSK-P-0064623-T01-IM7	*MSH6*	SNV	Q698*
GENIE-MSK-P-0061685	GENIE-MSK-P-0061685-T01-IM7	*MLH1*	SNV	S446Lfs*32
GENIE-MSK-P-0061685	GENIE-MSK-P-0061685-T01-IM7	*MSH2*	SNV	A230Lfs*16
GENIE-MSK-P-0067365	GENIE-MSK-P-0067365-T01-IM7	*MSH2*	SNV	G820Vfs*21
GENIE-MSK-P-0062736	GENIE-MSK-P-0062736-T01-IM7	*MSH2*	SNV	T441Nfs*2
TCGA-KK-A7B1	TCGA-KK-A7B1-01	*MLH1*	CNV	Homozygous deletion
TCGA-KK-A7AY	TCGA-KK-A7AY-01	*PMS2*	CNV	Homozygous deletion
TCGA-V1-A8X3	TCGA-V1-A8X3-01	*PMS2*	CNV	Homozygous deletion
GENIE-MSK-P-0029164	GENIE-MSK-P-0029164-T01-IM6	*MSH2, MSH6*	CNV	Homozygous deletion
GENIE-MSK-P-0029323	GENIE-MSK-P-0029323-T01-IM6	*MSH2, MSH6*	CNV	Homozygous deletion
GENIE-MSK-P-0030013	GENIE-MSK-P-0030013-T01-IM6	*MSH2, MSH6*	CNV	Homozygous deletion
GENIE-MSK-P-0031855	GENIE-MSK-P-0031855-T01-IM6	*MSH2, MSH6*	CNV	Homozygous deletion
GENIE-MSK-P-0035133	GENIE-MSK-P-0035133-T01-IM6	*MSH2*	CNV	Homozygous deletion
GENIE-MSK-P-0036805	GENIE-MSK-P-0036805-T01-IM6	*MSH2*	CNV	Homozygous deletion
GENIE-MSK-P-0039351	GENIE-MSK-P-0039351-T01-IM6	*MSH2, MSH6*	CNV	Homozygous deletion
GENIE-DFCI-169310	GENIE-DFCI-169310-2271502	*MSH2, MSH6*	CNV	Homozygous deletion
GENIE-MSK-P-0060405	GENIE-MSK-P-0060405-T01-IM7	*MSH2, MSH6*	CNV	Homozygous deletion
GENIE-MSK-P-0061209	GENIE-MSK-P-0061209-T01-IM7	*MSH6*	CNV	Homozygous deletion
GENIE-MSK-P-0062382	GENIE-MSK-P-0062382-T01-IM7	*MLH1*	SV	*MLH1*-intragenic
TCGA-KK-A5A1	TCGA-KK-A5A1-01	*MLH1*	SV	*MYRIP-MLH1* fusion
GENIE-MSK-P-0033077	GENIE-MSK-P-0033077-T01-IM6	*MSH2*	SV	*MSH2*-intragenic
GENIE-DFCI-195852	GENIE-DFCI-195852-2644796	*MSH2*	SV	*MSH2*-intragenic
GENIE-MSK-P-0024219	GENIE-MSK-P-0024219-T01-IM6	*MSH6*	SV	*MSH6-TTC7A* fusion

GENIE-MSK-P-0024219	GENIE-MSK-P-0024219-T01-IM6	*MSH6*	SV	*MSH6-TTC7A* fusion
GENIE-DFCI-112246	GENIE-DFCI-112246-526378	*MSH6*	SV	*MSH6*-intragenic

Supplementary Table S3. List of MMR-d metastatic tumors

Patient ID	Sample ID	Gene	Alteration Type	Alteration Description
GENIE-MSK-P-0000755	GENIE-MSK-P-0000755-T01-IM3	*MLH1*	SNV	R497Gfs*11
GENIE-MSK-P-0000755	GENIE-MSK-P-0000755-T01-IM3	*MSH2*	SNV	R621*
GENIE-MSK-P-0001230	GENIE-MSK-P-0001230-T01-IM3	*MLH1*	SNV	T148I
GENIE-MSK-P-0003665	GENIE-MSK-P-0003665-T01-IM5	*MSH2*	SNV	E48*
GENIE-MSK-P-0003665	GENIE-MSK-P-0003665-T01-IM5	*MSH6*	SNV	R361C
GENIE-MSK-P-0004910	GENIE-MSK-P-0004910-T01-IM5	*MSH2*	SNV	P591Q
GENIE-MSK-P-0004910	GENIE-MSK-P-0004910-T01-IM5	*MSH6*	SNV	F1104Lfs*11
GENIE-MSK-P-0005111	GENIE-MSK-P-0005111-T01-IM5	*PMS2*	SNV	Q275*
GENIE-DFCI-004876	GENIE-DFCI-004876-3827	*MSH2*	SNV	E562Q
GENIE-MSK-P-0012928	GENIE-MSK-P-0012928-T01-IM5	*PMS2*	SNV	Y268Tfs*39
GENIE-MSK-P-0015211	GENIE-MSK-P-0015211-T01-IM6	*MSH6*	SNV	F1104Lfs*11
GENIE-MSK-P-0008667	GENIE-MSK-P-0008667-T03-IM6	*MSH6*	SNV	F1088Lfs*5
GENIE-DFCI-011525	GENIE-DFCI-011525-11235	*MLH1*	SNV	X633_splice
GENIE-MSK-P-0017886	GENIE-MSK-P-0017886-T01-IM6	*MSH6*	SNV	I972Lfs*25
GENIE-MSK-P-0019937	GENIE-MSK-P-0019937-T01-IM6	*PMS2*	SNV	E582Rfs*8
GENIE-DFCI-077379	GENIE-DFCI-077379-299104	*PMS2*	SNV	S416C
GENIE-DFCI-037461	GENIE-DFCI-037461-130296	*MSH2*	SNV	E177*
GENIE-DFCI-037461	GENIE-DFCI-037461-130296	*MSH6*	SNV	R577H
GENIE-VICC-184903	GENIE-VICC-184903-unk-1	*MSH6*	SNV	S1340Tfs*7
GENIE-DFCI-096154	GENIE-DFCI-096154-663890	*PMS2*	SNV	F686C
GENIE-DFCI-098561	GENIE-DFCI-098561-571476	*MSH6*	SNV	T1225M
GENIE-DFCI-103001	GENIE-DFCI-103001-664168	*MLH1*	SNV	R18C
GENIE-DFCI-111090	GENIE-DFCI-111090-526543	*MSH6*	SNV	C783S
GENIE-MSK-P-0021894	GENIE-MSK-P-0021894-T02-IM6	*MSH2*	SNV	E786Dfs*11
GENIE-MSK-P-0024650	GENIE-MSK-P-0024650-T01-IM6	*MSH6*	SNV	K247Nfs*32
GENIE-MSK-P-0024686	GENIE-MSK-P-0024686-T01-IM6	*PMS2*	SNV	Q449*
GENIE-MSK-P-0029087	GENIE-MSK-P-0029087-T01-IM6	*MSH6*	SNV	R1076H
GENIE-MSK-P-0029248	GENIE-MSK-P-0029248-T01-IM6	*MSH6*	SNV	F1088Sfs*2
GENIE-MSK-P-0030831	GENIE-MSK-P-0030831-T01-IM6	*MSH6*	SNV	K1288N
GENIE-DFCI-097596	GENIE-DFCI-097596-757405	*MSH2*	SNV	A230Lfs*16
GENIE-DFCI-097596	GENIE-DFCI-097596-757405	*MSH6*	SNV	F1088Lfs*5
GENIE-DFCI-097311	GENIE-DFCI-097311-805372	*MSH6*	SNV	M492V
GENIE-MSK-P-0032701	GENIE-MSK-P-0032701-T02-IM6	*MSH2*	SNV	V3Dfs*74
GENIE-MSK-P-0036029	GENIE-MSK-P-0036029-T02-IM7	*MSH6*	SNV	Q572*
GENIE-DFCI-142788	GENIE-DFCI-142788-1358550	*MSH6*	SNV	F1088Lfs*5
GENIE-DFCI-142788	GENIE-DFCI-142788-1358550	*PMS2*	SNV	S46I
GENIE-DFCI-170500	GENIE-DFCI-170500-1525865	*MLH1*	SNV	R389W
GENIE-MSK-P-0043336	GENIE-MSK-P-0043336-T01-IM6	*MSH2*	SNV	Q324*
GENIE-MSK-P-0046590	GENIE-MSK-P-0046590-T02-IM6	*MSH2*	SNV	K393Ffs*18
GENIE-DFCI-153034	GENIE-DFCI-153034-2271469	*PMS2*	SNV	R802*

GENIE-DFCI-173945	GENIE-DFCI-173945-1903411	*PMS2*	SNV	Y310C
GENIE-DFCI-174496	GENIE-DFCI-174496-2021516	*PMS2*	SNV	X330_splice
GENIE-DFCI-186495	GENIE-DFCI-186495-2904280	*PMS2*	SNV	T728Afs*7
GENIE-DFCI-202219	GENIE-DFCI-202219-2970119	*MSH6*	SNV	F1088Lfs*5
GENIE-DFCI-256069	GENIE-DFCI-256069-3646077	*MLH1*	SNV	X347_splice
GENIE-MSK-P-0058939	GENIE-MSK-P-0058939-T02-IM7	*MSH6*	SNV	R495*
GENIE-MSK-P-0063582	GENIE-MSK-P-0063582-T01-IM7	*MSH6*	SNV	F1088Lfs*5, R1095H
GENIE-MSK-P-0061912	GENIE-MSK-P-0061912-T01-IM7	*MSH6*	SNV	V558M
GENIE-UCSF-13670	GENIE-UCSF-13670-3171T	*MSH6*	SNV	F1037L
GENIE-UCSF-14714	GENIE-UCSF-14714-1780T	*MLH1*	SNV	X102_splice
GENIE-UCSF-14919	GENIE-UCSF-14919-5905T	*MSH6*	SNV	X1334_splice
GENIE-MSK-P-0012930	GENIE-MSK-P-0012930-T01-IM5	*MLH1*	CNV	Homozygous deletion
GENIE-MSK-P-0008667	GENIE-MSK-P-0008667-T03-IM6	*MLH1*	CNV	Homozygous deletion
GENIE-MSK-P-0001774	GENIE-MSK-P-0001774-T01-IM3	*MSH2*	CNV	Homozygous deletion
GENIE-MSK-P-0008682	GENIE-MSK-P-0008682-T01-IM5	*MSH2, MSH6*	CNV	Homozygous deletion
GENIE-MSK-P-0012928	GENIE-MSK-P-0012928-T01-IM5	*MSH2, MSH6*	CNV	Homozygous deletion
GENIE-MSK-P-0019023	GENIE-MSK-P-0019023-T05-IM6	*MSH2, MSH6*	CNV	Homozygous deletion
GENIE-MSK-P-0019937	GENIE-MSK-P-0019937-T01-IM6	*MSH2, MSH6*	CNV	Homozygous deletion
GENIE-MSK-P-0025952	GENIE-MSK-P-0025952-T01-IM6	*MSH2, MSH6*	CNV	Homozygous deletion
GENIE-MSK-P-0027637	GENIE-MSK-P-0027637-T02-IM6	*MSH2, MSH6*	CNV	Homozygous deletion
GENIE-MSK-P-0029248	GENIE-MSK-P-0029248-T01-IM6	*MSH2*	CNV	Homozygous deletion
GENIE-MSK-P-0050196	GENIE-MSK-P-0050196-T01-IM6	*MSH2, MSH6*	CNV	Homozygous deletion
GENIE-DFCI-332769	GENIE-DFCI-332769-3960475	*MSH2*	CNV	Homozygous deletion
GENIE-MSK-P-0064150	GENIE-MSK-P-0064150-T01-IM7	*MSH2, MSH6*	CNV	Homozygous deletion
GENIE-MSK-P-0058939	GENIE-MSK-P-0058939-T02-IM7	*MSH6*	CNV	Homozygous deletion
GENIE-MSK-P-0063582	GENIE-MSK-P-0063582-T01-IM7	*MSH6*	CNV	Homozygous deletion
GENIE-DFCI-238006	GENIE-DFCI-238006-3307418	*MLH1*	SV	*MLH1*-intragenic
GENIE-MSK-P-0014756	GENIE-MSK-P-0014756-T01-IM6	*MSH2*	SV	*MSH2*-intragenic
GENIE-DFCI-011525	GENIE-DFCI-011525-11235	*MSH2*	SV	*MSH2*-intergenic
GENIE-MSK-P-0058939	GENIE-MSK-P-0058939-T02-IM7	*MSH2*	SV	*MSH2*-intragenic
GENIE-MSK-P-0060952	GENIE-MSK-P-0060952-T01-IM7	*MSH2*	SV	*MSH2*-intragenic
GENIE-MSK-P-0062080	GENIE-MSK-P-0062080-T01-IM7	*MSH2*	SV	*MSH2*-intragenic
GENIE-MSK-P-0061330	GENIE-MSK-P-0061330-T01-IM7	*MSH6*	SV	*MSH6*-intragenic
GENIE-DFCI-417258	GENIE-DFCI-417258-4381272	*MSH6*	SV	*MSH6*-intragenic

Supplementary Table S4. SNV frequency by MMR status in primary tumors

Gene	SNV No. MMR-d	SNV Freq. MMR-d	SNV No. MMR-p	SNV Freq. MMR-p	Nominal P.Value	Significant
JAK1	22	31.88	38	1.46	2.30E-21	Yes
ASXL1	20	28.99	27	1.04	5.62E-21	Yes
KMT2D	32	46.38	162	6.24	1.51E-19	Yes
RNF43	19	27.54	28	1.08	2.07E-19	Yes
SMARCA4	17	24.64	18	0.69	4.22E-19	Yes
NCOR1	20	28.99	38	1.46	8.72E-19	Yes
NF1	19	27.54	33	1.27	2.06E-18	Yes
POLD1	15	21.74	14	0.54	1.89E-17	Yes
SPEN	19	27.54	50	1.93	9.15E-16	Yes
KMT2A	19	27.54	51	1.97	1.23E-15	Yes
ZFHX3	23	33.33	93	3.58	1.29E-15	Yes
MSH3	12	17.39	11	0.42	3.67E-14	Yes
CIC	14	20.29	23	0.89	5.98E-14	Yes
FAT1	18	26.09	58	2.24	1.09E-13	Yes
POLE	14	20.29	29	1.12	6.82E-13	Yes
ATR	11	15.94	23	0.89	2.78E-10	Yes
KMT2C	19	27.54	127	4.89	1.51E-09	Yes
MGA	14	20.29	70	2.70	1.23E-08	Yes
PIK3CA	14	20.29	77	2.97	3.59E-08	Yes
ERBB4	9	13.04	24	0.92	7.26E-08	Yes
ERG	7	10.14	10	0.39	9.11E-08	Yes
CDK12	15	21.74	100	3.85	1.08E-07	Yes
ARID1B	11	15.94	49	1.89	1.96E-07	Yes
PTEN	18	26.09	157	6.05	1.98E-07	Yes
CREBBP	10	14.49	44	1.70	6.77E-07	Yes
ARID1A	10	14.49	51	1.97	2.21E-06	Yes
BRCA2	13	18.84	102	3.93	4.60E-06	Yes
RB1	8	11.59	37	1.43	1.35E-05	Yes
ATM	13	18.84	115	4.43	1.53E-05	Yes
BRIP1	6	8.70	18	0.69	2.26E-05	Yes
PMS1	5	7.25	10	0.39	2.47E-05	Yes
ETV1	5	7.25	10	0.39	2.47E-05	Yes
CHEK2	5	7.25	13	0.50	6.65E-05	Yes
NBN	4	5.80	8	0.31	0.000174566	Yes
IDH1	5	7.25	18	0.69	0.000235947	No
FANCA	5	7.25	20	0.77	0.000357831	No
AR	5	7.25	22	0.85	0.000522255	No
TMPRSS2	5	7.25	22	0.85	0.000522255	No
PIK3R1	5	7.25	25	0.96	0.000867893	No

MED12	7	10.14	57	2.20	0.001104276	No
KDM6A	8	11.59	86	3.31	0.002528629	No
PALB2	4	5.80	21	0.81	0.003458218	No
FOXA1	17	24.64	317	12.22	0.004825269	No
BRCA1	4	5.80	24	0.92	0.005279455	No
APC	9	13.04	128	4.93	0.007842192	No
MYC	3	4.35	20	0.77	0.020324323	No
BRAF	4	5.80	47	1.81	0.04131884	No
TP53	22	31.88	555	21.39	0.052609143	No
CDKN1B	3	4.35	35	1.35	0.073645057	No
RAD51C	1	1.45	2	0.08	0.075735202	No
CTNNB1	4	5.80	61	2.35	0.08573971	No
EPCAM	1	1.45	3	0.12	0.099701482	No
SPOP	12	17.39	331	12.76	0.272157039	No
MUTYH	1	1.45	12	0.46	0.289602119	No

Supplementary Table S5. SNV frequency by MMR status in metastatic tumors

Gene	SNV No. MMR-d	SNV Freq. MMR-d	SNV No. MMR-p	SNV Freq. MMR-p	Nominal P.Value	Significant
JAK1	17	28.33	34	2.52	2.39E-12	Yes
ERBB4	12	20.00	20	1.48	1.36E-09	Yes
KMT2A	12	20.00	28	2.08	2.59E-08	Yes
KMT2D	20	33.33	103	7.64	3.21E-08	Yes
CIC	11	18.33	24	1.78	6.21E-08	Yes
FAT1	13	21.67	44	3.26	2.35E-07	Yes
SPEN	11	18.33	30	2.22	3.86E-07	Yes
RNF43	10	16.67	24	1.78	5.42E-07	Yes
CREBBP	10	16.67	25	1.85	7.34E-07	Yes
BRCA1	8	13.33	13	0.96	8.91E-07	Yes
SMARCA4	9	15.00	19	1.41	9.20E-07	Yes
MGA	12	20.00	49	3.63	4.05E-06	Yes
POLE	9	15.00	27	2.00	9.59E-06	Yes
ERG	5	8.33	5	0.37	2.54E-05	Yes
ARID1B	8	13.33	24	1.78	3.18E-05	Yes
ASXL1	10	16.67	44	3.26	5.07E-05	Yes
AR	16	26.67	129	9.56	0.000192646	No
KMT2C	12	20.00	76	5.63	0.000195135	No
NBN	5	8.33	10	0.74	0.000256266	No
ETV1	4	6.67	9	0.67	0.001594324	No
APC	14	23.33	128	9.49	0.001705111	No
FANCA	5	8.33	18	1.33	0.002205235	No
BRCA2	9	15.00	63	4.67	0.002576811	No
NCOR1	5	8.33	20	1.48	0.003260111	No
ZFHX3	9	15.00	66	4.89	0.003435638	No
PMS1	3	5.00	6	0.44	0.005137611	No
ATR	4	6.67	15	1.11	0.007129477	No
ARID1A	6	10.00	37	2.74	0.008235443	No
MSH3	3	5.00	8	0.59	0.009493499	No
ATM	9	15.00	88	6.52	0.018566625	No
CHEK2	3	5.00	11	0.82	0.01911636	No
MYC	3	5.00	11	0.82	0.01911636	No
NF1	4	6.67	24	1.78	0.028278698	No
POLD1	3	5.00	16	1.19	0.043755968	No
MED12	4	6.67	29	2.15	0.048259618	No
PALB2	3	5.00	18	1.33	0.056558955	No
CDK12	8	13.33	87	6.45	0.057687413	No
PTEN	10	16.67	120	8.90	0.062708181	No
RB1	6	10.00	62	4.60	0.064628669	No

BRIP1	2	3.33	9	0.67	0.076610608	No
RAD51D	1	1.67	1	0.07	0.083382396	No
BRAF	3	5.00	25	1.85	0.11313236	No
PIK3R1	4	6.67	40	2.97	0.113509245	No
TMPRSS2	2	3.33	14	1.04	0.146122942	No
EPCAM	1	1.67	4	0.30	0.195793847	No
PIK3CA	5	8.33	69	5.11	0.239627519	No
MUTYH	1	1.67	6	0.44	0.263084156	No
RAD51C	1	1.67	6	0.44	0.263084156	No
CDKN1B	2	3.33	25	1.85	0.320668534	No
IDH1	1	1.67	8	0.59	0.324830152	No
CTNNB1	5	8.33	81	6.00	0.408347896	No
TP53	20	33.33	503	37.29	0.586716003	No
KDM6A	2	3.33	43	3.19	0.717235558	No
SPOP	7	11.67	146	10.82	0.831428903	No
FOXA1	8	13.33	171	12.68	0.843213187	No

Supplementary Table S6. CNV frequency by MMR status in primary tumors

Homozygous Deletion

**Gene**	**CNV No. MMR-d**	**CNV Freq. MMR-d**	**CNV No. MMR-p**	**CNV Freq. MMR-p**	**Nominal P.Value**	**Significant**
EPCAM	7	10.60606	0	0	4.81E-12	YES
EPAS1	4	8	1	0.047506	1.26E-06	YES
ETV1	2	3.030303	2	0.078647	0.00366	NO
KMT2C	2	3.921569	4	0.188058	0.007595	NO
AGO2	0	0	1	0.047506	1	NO
ANKRD11	2	4	30	1.425178	0.168836	NO
APC	0	0	16	0.623782	1	NO
AR	0	0	0	0	1	NO
ATM	0	0	10	0.389864	1	NO
ATR	0	0	2	0.077973	1	NO
BRAF	0	0	0	0	1	NO
BRCA1	0	0	6	0.233918	1	NO
BRCA2	0	0	32	1.247563	1	NO
BRIP1	0	0	0	0	1	NO
CDK12	0	0	2	0.077973	1	NO
CDKN1B	1	1.492537	48	1.871345	1	NO
CHEK2	0	0	9	0.350877	1	NO
CTNNB1	0	0	1	0.038986	1	NO
CXCR4	2	3.030303	36	1.415651	0.249617	NO
CYSLTR2	2	4	57	2.707838	0.400578	NO
ERF	0	0	19	0.902613	1	NO
ERG	1	1.492537	48	1.871345	1	NO
FANCA	3	4.477612	30	1.169591	0.049944	NO
FOXA1	0	0	1	0.039324	1	NO
FOXF1	2	4	23	1.092637	0.112893	NO
FOXO1	2	4	43	2.042755	0.280698	NO
HOXB13	0	0	0	0	1	NO
IDH1	0	0	2	0.077973	1	NO
KMT2D	0	0	0	0	1	NO
MED12	0	0	1	0.039324	1	NO
MSH3	1	1.960784	12	0.564175	0.265739	NO
MUTYH	0	0	2	0.077973	1	NO
MYC	0	0	2	0.077973	1	NO
NBN	0	0	2	0.077973	1	NO
NCOR1	1	2	2	0.095012	0.068034	NO
PALB2	0	0	0	0	1	NO
PIK3CA	0	0	5	0.194932	1	NO

PLCG2	2	4	34	1.615202	0.202621	NO
PMS1	0	0	0	0	1	NO
POLD1	0	0	2	0.077973	1	NO
POLE	0	0	8	0.311891	1	NO
PRDM14	0	0	0	0	1	NO
PREX2	0	0	0	0	1	NO
PTEN	3	4.477612	211	8.226121	0.365469	NO
RAD51C	0	0	4	0.157295	1	NO
RAD51D	0	0	3	0.117971	1	NO
RB1	2	2.985075	67	2.612086	0.694849	NO
RECQL4	0	0	0	0	1	NO
RNF43	0	0	8	0.311891	1	NO
SPOP	0	0	1	0.038986	1	NO
TBX3	0	0	5	0.235073	1	NO
TCEB1	0	0	0	0	1	NO
TMPRSS2	1	1.515152	62	2.438065	1	NO
TP53	1	1.492537	31	1.208577	0.563994	NO
ZFHX3	3	6	42	1.995249	0.084432	NO

Supplementary Table S7. CNV frequency by MMR status in metastatic tumors

Homozygous Deletion

**Gene**	**CNV No. MMR-d**	**CNV Freq. MMR-d**	**CNV No. MMR-p**	**CNV Freq. MMR-p**	**Nominal P.Value**	**Significant**
EPCAM	12	21.42857	0	0	7.87E-18	YES
EPAS1	2	5.714286	1	0.094877	0.002952	NO
INHA	2	5.714286	3	0.28463	0.009447	NO
AGO2	0	0	2	0.189753	1	NO
AMER1	1	2.857143	2	0.189753	0.093436	NO
ANKRD11	1	2.857143	37	3.510436	1	NO
APC	0	0	26	1.984733	0.622891	NO
AR	0	0	1	0.076336	1	NO
ATM	0	0	7	0.534351	1	NO
ATR	0	0	0	0	1	NO
BRAF	0	0	1	0.076336	1	NO
BRCA1	0	0	2	0.152672	1	NO
BRCA2	1	1.754386	43	3.282443	1	NO
BRIP1	0	0	0	0	1	NO
CDK12	0	0	1	0.076336	1	NO
CDKN1B	1	1.754386	57	4.351145	0.510976	NO
CHEK2	0	0	4	0.305344	1	NO
CTNNB1	0	0	0	0	1	NO
ERF	0	0	10	0.948767	1	NO
ERG	0	0	1	0.076336	1	NO
ETV1	0	0	3	0.232378	1	NO
FANCA	3	5.263158	40	3.053435	0.420695	NO
FGF19	0	0	1	0.093197	1	NO
FGFR1	1	1.754386	28	2.137405	1	NO
FOXA1	0	0	1	0.077459	1	NO
HOXB13	0	0	0	0	1	NO
IDH1	0	0	0	0	1	NO
KMT2C	0	0	4	0.372787	1	NO
KMT2D	0	0	1	0.077459	1	NO
MAP3K1	2	3.508772	37	2.824427	0.675947	NO
MED12	0	0	2	0.154919	1	NO
MSH3	0	0	6	0.55918	1	NO
MUTYH	0	0	1	0.076336	1	NO
MYC	0	0	0	0	1	NO
NBN	0	0	0	0	1	NO
NCOR1	0	0	8	0.759013	1	NO
PALB2	0	0	3	0.229008	1	NO

PIK3CA	0	0	0	0	1	NO
PLK2	1	2.857143	30	2.8463	1	NO
PMS1	1	1.785714	2	0.154919	0.119693	NO
POLD1	0	0	1	0.076336	1	NO
POLE	0	0	5	0.381679	1	NO
PTEN	7	12.2807	230	17.55725	0.373257	NO
RAD51C	0	0	1	0.077459	1	NO
RAD51D	0	0	0	0	1	NO
RB1	1	1.754386	74	5.648855	0.365345	NO
RECQL4	0	0	1	0.077459	1	NO
RNF43	0	0	4	0.305344	1	NO
SPOP	0	0	1	0.076336	1	NO
TCEB1	0	0	0	0	1	NO
TMPRSS2	0	0	15	1.16189	1	NO
TP53	2	3.508772	44	3.358779	0.717493	NO
ZFHX3	1	2.857143	41	3.889943	1	NO

Supplementary Table S8. CNV frequency by MMR status in primary tumors

High level of amplification

**Gene**	**CNV No. MMR-d**	**CNV Freq. MMR-d**	**CNV No. MMR-p**	**CNV Freq. MMR-p**	**Nominal P.Value**	**Significant**
BRAF	1	1.492537	8	0.311891	0.207393	NO
TBX3	2	3.921569	42	1.974612	0.275518	NO
AGO2	2	4	55	2.612827	0.383884	NO
TMPRSS2	1	1.515152	21	0.825796	0.432211	NO
RECQL4	2	3.030303	67	2.634683	0.693319	NO
ANKRD11	0	0	1	0.047506	1	NO
APC	0	0	1	0.038986	1	NO
AR	0	0	34	1.325536	1	NO
ATM	0	0	3	0.116959	1	NO
ATR	0	0	10	0.389864	1	NO
BRCA1	0	0	3	0.116959	1	NO
BRCA2	0	0	2	0.077973	1	NO
BRIP1	0	0	8	0.311891	1	NO
CDK12	0	0	3	0.116959	1	NO
CDKN1B	0	0	6	0.233918	1	NO
CHEK2	0	0	1	0.038986	1	NO
CTNNB1	0	0	2	0.077973	1	NO
CXCR4	0	0	0	0	1	NO
CYSLTR2	0	0	0	0	1	NO
EPAS1	0	0	2	0.095012	1	NO
EPCAM	0	0	1	0.039324	1	NO
ERF	0	0	0	0	1	NO
ERG	0	0	1	0.038986	1	NO
ETV1	0	0	9	0.353913	1	NO
FANCA	0	0	1	0.038986	1	NO
FOXA1	1	1.515152	57	2.241447	1	NO
FOXF1	0	0	0	0	1	NO
FOXO1	0	0	0	0	1	NO
HOXB13	0	0	19	0.747149	1	NO
IDH1	0	0	1	0.038986	1	NO
KMT2C	0	0	5	0.235073	1	NO
KMT2D	0	0	1	0.039324	1	NO
MED12	0	0	1	0.039324	1	NO
MSH3	0	0	1	0.047015	1	NO
MUTYH	0	0	0	0	1	NO
MYC	2	2.985075	95	3.703704	1	NO
NBN	1	1.492537	65	2.534113	1	NO

NCOR1	0	0	1	0.047506	1	NO
PALB2	0	0	5	0.194932	1	NO
PIK3CA	0	0	16	0.623782	1	NO
PLCG2	0	0	0	0	1	NO
PMS1	0	0	0	0	1	NO
POLD1	0	0	2	0.077973	1	NO
POLE	0	0	2	0.077973	1	NO
PRDM14	1	2	69	3.27791	1	NO
PREX2	1	1.960784	58	2.726845	1	NO
PTEN	0	0	2	0.077973	1	NO
RAD51C	0	0	1	0.039324	1	NO
RAD51D	0	0	2	0.078647	1	NO
RB1	0	0	0	0	1	NO
RNF43	0	0	1	0.038986	1	NO
SPOP	0	0	4	0.155945	1	NO
TCEB1	1	2	73	3.467933	1	NO
TP53	0	0	0	0	1	NO
ZFHX3	0	0	0	0	1	NO

Supplementary Table S9. CNV frequency by MMR status in metastatic tumors

High level of amplification

**Gene**	**CNV No. MMR-d**	**CNV Freq. MMR-d**	**CNV No. MMR-p**	**CNV Freq. MMR-p**	**Nominal P.Value**	**Significant**
NBN	0	0	71	5.419847	0.069624	NO
TCEB1	0	0	70	6.641366	0.162256	NO
MYC	3	5.263158	131	10	0.359574	NO
RECQL4	1	1.785714	68	5.267235	0.359606	NO
FOXA1	0	0	23	1.781565	0.620926	NO
FGFR1	0	0	29	2.21374	0.62994	NO
AR	14	24.5614	365	27.8626	0.652452	NO
AGO2	2	5.714286	47	4.459203	0.668541	NO
AMER1	2	5.714286	64	6.072106	1	NO
ANKRD11	0	0	0	0	1	NO
APC	0	0	0	0	1	NO
ATM	0	0	1	0.076336	1	NO
ATR	0	0	14	1.068702	1	NO
BRAF	0	0	5	0.381679	1	NO
BRCA1	0	0	2	0.152672	1	NO
BRCA2	0	0	2	0.152672	1	NO
BRIP1	0	0	6	0.458015	1	NO
CDK12	0	0	2	0.152672	1	NO
CDKN1B	0	0	1	0.076336	1	NO
CHEK2	0	0	0	0	1	NO
CTNNB1	0	0	1	0.076336	1	NO
EPAS1	0	0	0	0	1	NO
EPCAM	0	0	0	0	1	NO
ERF	0	0	0	0	1	NO
ERG	0	0	3	0.229008	1	NO
ETV1	0	0	7	0.542215	1	NO
FANCA	0	0	0	0	1	NO
FGF19	1	2.777778	30	2.795899	1	NO
HOXB13	0	0	13	1.006971	1	NO
IDH1	0	0	0	0	1	NO
INHA	0	0	1	0.094877	1	NO
KMT2C	0	0	2	0.186393	1	NO
KMT2D	0	0	0	0	1	NO
MAP3K1	0	0	0	0	1	NO
MED12	0	0	20	1.549187	1	NO
MSH3	0	0	0	0	1	NO
MUTYH	0	0	1	0.076336	1	NO

NCOR1	0	0	2	0.189753	1	NO
PALB2	0	0	3	0.229008	1	NO
PIK3CA	0	0	3	0.229008	1	NO
PLK2	0	0	1	0.094877	1	NO
PMS1	0	0	2	0.154919	1	NO
POLD1	0	0	0	0	1	NO
POLE	0	0	0	0	1	NO
PTEN	0	0	0	0	1	NO
RAD51C	0	0	5	0.387297	1	NO
RAD51D	0	0	1	0.077459	1	NO
RB1	0	0	0	0	1	NO
RNF43	0	0	4	0.305344	1	NO
SPOP	0	0	1	0.076336	1	NO
TMPRSS2	0	0	3	0.232378	1	NO
TP53	0	0	0	0	1	NO
ZFHX3	0	0	0	0	1	NO
